# Adaptation of a Health Education Program for Improving the Uptake of HIV Self-Testing by Men in Rwanda: A Study Protocol

**DOI:** 10.3390/medicina56040149

**Published:** 2020-03-26

**Authors:** Tafadzwa Dzinamarira, Tivani Phosa Mashamba-Thompson

**Affiliations:** 1 Department of Public Health Medicine, School of Nursing and Public Health, University of KwaZulu-Natal, Durban 4001, South Africa; 2 CIHR Canadian HIV Trials Network, Vancouver, BC V6Z 1Y6, Canada; 3 Department of Public Health, University of Limpopo, Polokwane, Limpopo Province 0727, South Africa

**Keywords:** health education program, men, HIV self-testing

## Abstract

*Background and objectives*: Available evidence shows a low uptake of HIV services among men in Rwanda. HIV self-testing (HIVST), a new intervention, may have the potential to improve the uptake of HIV testing services among men. The current study aims to adapt a health education program (HEP) for improving the uptake of HIVST among men in Rwanda. *Materials and Methods*: We propose a mixed method study, which will be conducted in four phases. In Phase 1, we will conduct a scoping review to map the available evidence on health education programs for men in low- and middle-income countries (LMICs). In Phase 2, we will conduct interviews with stakeholders in the Rwanda HIV response and healthcare providers to determine their perspectives on implementation of HIVST in Rwanda. In Phase 3, a cross-sectional survey will be used to assess HIVST awareness and acceptability among men in Rwanda. Guided by findings from Phases 1, 2, and 3, we will employ the nominal group technique to develop and optimize the HEP for improving the uptake of HIVST among men in Rwanda. In Phase 4, we will use a pragmatic pilot randomized controlled trial to assess the preliminary impact of the HEP for men in Rwanda and assess the feasibility of a later, larger study. We will employ the Stata version 16 statistical package and NVivo version 12 for the analysis of quantitative and qualitative data, respectively. We anticipate that the findings of this study will inform implementers and policy makers to guide strategies on the implementation of HIVST in Rwanda and ultimately accomplish goals set forth in the Rwanda 2019–2024 Fourth Health Sector Strategic Plan on scaling up the number of men who know their HIV status. *Conclusion:* It is anticipated that this study will proffer solutions and strategies that are applicable not only in Rwanda but also in similar settings of LMICs.

## Contributions to the Literature

- Policy development for the implementation of HIV self-testing varies from country to country, indicating that the implementation strategies would differ accordingly.
- The current study will utilize a pragmatic approach to adapt and optimize a health education program that may be applicable to the local context but also proffer solutions for similar low- to middle-income country (LMIC) settings.
- Currently, there is a paucity of evidence on health education programs (HEPs) targeted for men specifically for HIV self-testing uptake.

## 1. Background

Human immunodeficiency virus (HIV) currently remains a major public health problem in Rwanda. In 2014, the Joint United Nations Programme on HIV/AIDS (UNAIDS) and partners launched the 90-90-90 targets for 2020 that call for 90% of people with HIV to be diagnosed, 90% of the diagnosed to access ART, and 90% of the treated to be virally suppressed [1]. Worldwide, different interventions that aim to scale up HIV diagnosis, antiretroviral therapy (ART) initiation, and care continuum have led to most countries nearing epidemic control [2,3]. However, available evidence shows a low uptake of HIV testing services (HTSs) among men [4]. 

The first documented AIDS cases in Rwanda were in 1983 [5]. Rwanda has the 18th highest HIV prevalence in the world [3]. Since 2005, HIV prevalence in Rwanda has been constant at around 3% [6]. Specifically for the age group 15–49, HIV prevalence in Rwanda has disparities by sex, with 4% and 2% among women and men, respectively [6]. As of 2017, UNAIDS reported Rwanda to be nearing epidemic control for HIV with a steady decrease in HIV incidence (from 12,500 new cases in 2007 to 5000 in 2017) and AIDS deaths (from 12,500 in 2007 to 10,000 in 2017) [3,7,8,9]. A low uptake of HTSs remains a public health priority in Rwanda [6]. Findings from a recently concluded population-based HIV impact assessment study revealed that only 80.4% of HIV-positive men knew their HIV status [10]. This was much lower to their women counterparts; 85.6% of HIV-positive women knew their HIV status [10]. This underscores the need to improve the first 90 among men. In response to this, one of the strategies highlighted in the Rwanda Ministry of Health 2019–2024 Fourth Health Sector Strategic Plan is the scaling-up of male-targeted interventions with the main focus on increasing the uptake of HTSs [11]. 

Epidemic control of HIV infection depends on the success of the strategies to prevent new infections and increase the treatment availability and uptake for currently infected individuals [2,12]. For prevention and treatment interventions, the importance of knowing one’s HIV status cannot be over-emphasized. While this is the case, men (ranging between 51% and 70%) lag behind women (ranging between 71% and 84%) on the uptake of HTSs, a gap threatening the progress towards the 90-90-90 goals [3,4,13]. A poor uptake of HTSs among men has been attributed to patriarchy and masculinity [14,15]. These sociocultural factors refer to the socially determined stereotypes, which are infringed on men as acceptable “manliness”. These cultural and traditional masculine identities have been constructed by the societies and often inform men’s health seeking behavior [14]. Poor service delivery, long waiting times to obtain the results, concerns over confidentiality, and HIV related-stigma have been reported elsewhere as impeding men’s uptake of HTSs [13,15,16]. The World Health Organization (WHO) published the first global guidelines on HIV self-testing (HIVST) in 2016 [17]—an intervention that has shown global potential to increase uptake of HTSs [13,18,19,20,21]. Studies have reported success, with HIVST being offered to men in health care facility-based settings [22,23] and community settings [22], in Malawi, Zambia, and Zimbabwe. An HIVST scale-up study conducted in these three African countries revealed promising results. A higher proportion of male self-testers (22.3%), compared with women (17.1%), were first-time testers, which was apparent in Zimbabwe (16.2% vs. 11.4%), Zambia (25.4 vs. 17.7%), and Malawi (27.9 vs. 25.9%) [22]. Several other different scholars have shown HIVST as an acceptable strategy to improve men’s uptake of HTSs [16,24,25,26]. In the Rwandan context, HIVST may have potential to address men’s barriers of HTSs associated with facility-based testing [24,27,28]. In Rwanda, a lack of awareness on HIVST has been revealed as an important barrier to uptake [28]. 

Historically, health education programs (HEPs) have been shown to play an important role in not only the provision of knowledge to individuals concerning their health but also building skills and changing attitudes regarding health issues [29]. Given that the factors that contribute to male aversion to HTSs are multifaceted, they require equally complex interventions [30]. The primary aim of this study will be an adaptation of an HEP for improving the uptake of HIVST by men in Rwanda. It is anticipated that the results of this will proffer solutions and strategies that are applicable for men in similar settings of low- to middle-income countries (LMICs).

### 1.1. Ethical Considerations

This study has been ethically reviewed and approved by four institutional review boards: The Rwanda National Ethics Committee (Approval number: 332/RNEC/201), the University Teaching Hospital of Kigali Ethics Committee (Approval number: EC/CHUK/0111/2019), Rwanda Military Hospital Institutional Review Board (Approval number: RMH IRB/036/2019) and the University of KwaZulu Natal Biomedical Research Ethics Committee (Approval number: BE/280/19). All participants will sign a consent form (Additional File) prior to participation in the study.

### 1.2. Trial Registration

Pan African Clinical Trial Registry, PACTR201908758321490), Registered 8 August 2019, https://pactr.samrc.ac.za/TrialDisplay.aspx?TrialID=8310.

## 2. Methods

### 2.1. Study Design

Guided by the Standard Protocol Items: Recommendations for Interventional Trials (SPIRIT), we propose a multi-phase mixed method study. In Phase 1, we will conduct a scoping review to map available evidence on HEPs for men in LMICs. We chose to use the scoping methodology, a useful approach for determining the need for, and value of, a full systematic review. In Phase 2, we will conduct interviews with stakeholders in the Rwanda HIV response and healthcare providers to determine their perspectives on the implementation of HIVST in Rwanda. In Phase 3, a cross-sectional survey will be used to assess HIVST awareness among men in Rwanda.

Guided by findings from Phases 1, 2, and 3, we will employ the nominal group technique [31] to co-create and optimize the HEP for improving the uptake of HIVST among men in Rwanda. In Phase 4, we will use a pragmatic pilot randomized controlled trial (RCT) to assess the feasibility of a larger trial and the preliminary impact of the HEP.

### 2.2. Theoretical Framework

Two models will guide this study. The Consolidated Framework for Implementation Research (CFIR) [32] and the Theory of Change (ToC) [33]. The CFIR will frame the overall project design across methods. This framework was adapted to suit the study. The CFIR provides a menu of constructs that can be used in a range of applications [32]. The domains of the framework include intervention characteristics, an inner setting, an outer setting, the characteristics of individuals involved in the implementation, and the implementation process [32]. All these domains affect an intervention’s implementation. The process flow for development and optimization of HEPs will be guided by the CFIR. To adapt and optimize the HEP, a co-creation workshop will be done. A literature review by Greenhalgh et al. [34] revealed co-creation models to be an increasingly popular approach to aligning research and service development with the potential to deliver significant societal impact via dynamic, locally adaptive community-expert partnerships. A co-creation workshop of the community-based participatory research model [35] will be employed. This model has been shown to be useful when power imbalances between researchers and community members exist [35]. 

This study will be guided by the theory of change (ToC) [33]. The theory of change is a useful methodology for planning, participation, and evaluation applied to initiatives that are implemented to drive social change [36]. The main domains for a ToC are the inputs, outputs, outcomes, and impact [33,36,37]. Although ToCs come in different variations, there are key components that remain the same in each model. They begin with the determination of a final outcome and then map backwards from this goal towards the conditions needed to reach this goal [37]. A ToC is most useful in the areas of monitoring and evaluations of programs designed for significant social impact [38]; however, under this umbrella could be many other designated areas that include policy making [33]. Specifically, for the purposes of this study, the ToC will drive the individual-level HIVST intervention to be piloted in Phase 4. 

### 2.3. Study Setting

Kigali City Province is the capital city of Rwanda. There are three districts, namely Gasabo, Kicukiro, and Nyarugenge, 35 sectors, 161 cells, and 1183 villages in Kigali [39]. The population of Kigali was 1,132,686, with an estimated urban population of 859,332, as of 2012 [39]. At this time, the majority of the residents were male (51.7%) [39]. There are a total of 42 health facilities in Kigali; 21 in Gasabo, 10 in Kicukiro, and 11 in Nyarugenge [39]. HIV prevalence in Kigali has disparities by sex, with 8.0% among women and 4.4% among men. However, at the national level, HIV prevalence is 3.6% and 2.2% among women and men, respectively [6]. Among men across the three districts, HIV prevalence was reported to be 6.0% in Nyarugenge, 4.9% in Kicukiro, and 4.4% in Gasabo [6].

### 2.4. Phase 1: Scoping Review 

Objective 1: To conduct a scoping review on HEPs for men’s engagement in health services in LMICs.

Design: The scoping review protocol has been developed a priori and has been published elsewhere [40]. The scoping review will be reported according to the PRISMA Extension for Scoping Reviews (PRISMA-ScR) guidelines [41]. 

Data Source: Eligible studies will be selected guided by an inclusion and exclusion criteria. 

Analysis: We will use NVivo version 12 to extract relevant outcomes and thematic analysis of the studies.

Outcome Measures: A narrative report of evidence on HEPs for men’s engagement in health services in LMICs. 

### 2.5. Phase 2: Qualitative Study

Objective 2: To determine key stakeholders’ perceptions on the implementation of HIVST in Rwanda.

Design: A qualitative study design will be employed [42]. We will purposively sample and enroll a minimum of eight key stakeholders based on the researcher’s judgment that they would contribute valuable insights on HIVST implementation in Rwanda. An interview guide will be used to guide the discussion (Supplementary File 1). The interview guide will probe for information on the current status of HIVST implementation in Rwanda, the perceived challenges, and strategies for implementation and scale-up. All interviews will be tape-recorded. We will transcribe verbatim all tape-recorded interviews. Where applicable, the transcribed text from each respondent will be translated from Kinyarwanda, the local language, to English.

Data Source: Data will be gathered through in depth-interviews.

Analysis: Thematic content analysis will be based on the naturalistic paradigm [43] following the hybrid approach [44].

Outcome measures: A narrative report of key stakeholder and health care providers perspectives on the implementation of HIVST in Rwanda. Perspectives will be assessed using the intervention characteristics domains of the CFIR (Table 1).

### 2.6. Phase 3: Cross Sectional Survey

#### 2.6.1. Objective 3: To assess HIVST Awareness among Men in Kigali, Rwanda

Design: A cross-sectional study will be employed. One health facility will be selected in each district. Due to the nature of the study design, and assuming that the population is >10,000 and that there is an unknown level of awareness of HIVST among men in Rwanda, the overall sample size will be deduced using the formula below:

(1) $$n=\frac{Z^{2}\times \;\mathrm{pq}\;}{d^{2}}$$

(2) $$n=\frac{1.96^{2}\times \left(0.5\right)\times \left(1-0.5\right)}{0.05^{2}}$$

where *n* = 384 participants.

For HIVST acceptability, assuming the acceptability rate of 94% reported in Kenya [45], the calculated sample size based on the formula above is 241 participants. For the purposes of this study, the desired sample size will be the highest of the two outcome variables. Sample size will not be adjusted for design effect as we will purposively select one tertiary facility in each district hence limiting the issue of error commitment during the recruitment. To determine the minimum sample size per facility, the probability proportionate to size will be used. A sampling frame for each study site will be developed based on the number of the out-patient departments’ male clinic attendees. Table 2 illustrates how sample size will be calculated for each study site and sampling strategy. Enrollment of participants will be by systematic random sampling. All men visiting the facility for the following reasons will be eligible to participate for the study (accompanying someone, visiting someone, or seeking health services). Healthcare workers will be excluded as they are considered implementers of HIVST thus expected to be knowledgeable on the intervention.

Data collection will be conducted using a self-administered semi-structured questionnaire (Supplementary File 2). The questionnaire will address demographic characteristics, health status and sexual behavior, health seeking behavior, and knowledge, attitudes, and perceptions (KAPs) toward HIVST.

Data Source: A semi-structured questionnaire will be employed.

Analysis: Quantitative variables will be summarized by means and standard deviation. Qualitative variables will be summarized using frequency distribution. We will use the student t-test or the chi-square test to determine study variables associated with age awareness and the acceptability of HIVST. Univariate binary logistic regression analysis will be carried out to obtain preliminary insight into the unconditional association of each independent variable and dependent variables (awareness and acceptability). Multiple logistic regression will be employed to determine explanatory variables associated with awareness or acceptability status while adjusting for other study variables. All statistical tests will be concluded at a 5% level of significance employing Stata version 16 statistical package.

Outcome measures: We will assess two outcome measures: awareness and acceptability. Awareness will be considered as a yes response to the question “Do you know what HIVST is?”. Acceptability will be assessed by the question “As of 20 February 2017, it is recommended by the MOH to use HIVST kits as an additional strategy to reach out to people who are not yet tested, and there are plans for a national roll-out. Do you agree with this recommendation?”

#### 2.6.2. Objective 4: To develop and Optimize an HEP for Improving the Uptake of HIVST among Men in Rwanda

Design: Using the nominal group technique, purposive sampling will be done to recruit group participants. Individuals will be selected based on the researcher’s assessment that they will contribute to the adaptation of the HEP. A maximum of eight people is anticipated. Two staff from the HIV/AIDS and STIs Diseases Division of the Rwanda Biomedical Center at the national level, one representative each of a public and private health facility HIV department working in health promotion, and four representatives of the target population (men), purposively selected by the researcher. The principal investigator (first author) will serve as the convener and facilitator for the group. Guided by findings from the scoping review, by a qualitative study on the perspectives of key stakeholders, and by a cross-sectional survey on awareness and acceptability among men, information will be gathered by asking panel members to respond to questions posed by the moderator, and then by asking participants to prioritize the ideas or suggestions of each group member. The process is expected to prevent the domination of the discussion by a single person, encourage all group members to participate, and result in a set of prioritized strategies or recommendations that represent the group’s preferences for the HEP. Steps to be followed include introductions and explanations, a silent generation of ideas, sharing ideas, group discussion, voting, and ranking [31]. The increased number of heterogeneous inputs is expected to lead to quality decisions on key messages to prioritize for the HEP.

### 2.7. Recruitment Strategy

The principal investigator’s institution will send invitation letters to two purposively selected staff from the HIV/AIDS and STIs Diseases Division of the Rwanda Biomedical Center at the national level and one representative each of a public and private health facility HIV department working in health promotion. We will consult sector and cell leaders to enroll four representatives of the target population (men).

### 2.8. Inclusion Criteria

For this objective, we will enroll personnel employed by HIV/AIDS and STIs Diseases Division at the Rwanda Biomedical Center at the national level, or personnel employed by a public and private hospital and responsible for HIV services and health promotion as subject matter experts. Men over the age of 18 residing in Nyarugenge will be eligible as representatives of the target population.

Data Source: Data will be obtained from the transcripts.

Analysis: We will audiotape and transcribe the nominal group meeting. We will group related ideas to form themes. The themes will be ranked and prioritized. The ranking score will be between one and five, one being the least severe and five being the most severe barrier to the uptake of current HTSs. Thereafter, stakeholders will discuss key messages to include in the HEP to address each barrier. The panel members will receive a summary of the formulated HEP for validation.

Outcome: An adapted and optimized HEP tailored implementation strategy, likely to be effective for improving the uptake of HIVST among men in Rwanda, will be designed.

### 2.9. Phase 4: Pilot Randomized-Controlled Trial

Objective 5: HIVST was officially introduced on World AIDS Day in 2017 in Rwanda [46]. Since then, HIVST guidelines have been included in the national HIV prevention and management guidelines as of 1 July, 2018 [47]. Available evidence show a lack of awareness of HIVST among men in Kigali [28]. In this phase, we will aim to determine the preliminary effectiveness of the HEP on the uptake of HIVST among men in Rwanda and assess the feasibility of conducting a larger trial.

Design: The pilot RCT protocol is registered with the Pan African Clinical Trial Registry (Registration: PACTR201908758321490). We will conduct a two-arm pilot RCT. Participants will be randomized to the HEP arm or the control arm. In the control group, routine health education will be administered. In the intervention group, the adapted HEP will be administered in addition to routine health education. Data collection will be through interviewer-administered questionnaire captured onto mobile tablet devices using pre-programmed study software Open Data Kit.

Recruitment strategy: The University Teaching Hospital of Kigali (CHUK) will be purposively selected to pilot the implementation of the HEP intervention. CHUK is the largest hospital located in the district of Nyarugenge, Kigali. It is also the largest referral hospital in the country with a capacity of 519 beds. In 2017, the hospital recorded 140,559 patients with an average of 11,713 per month [11]. The PI of the study will introduce the intervention in the out-patients’ department, as these individuals are not expected to have critical conditions that would affect their ability to take part in the HEP or the interviewer-administered questionnaire. Trained health care professionals will administer the HEP to eligible participants. In this study, we will include participants who fit the following criteria:

- is an adult man 18 years and older;
- does not know his HIV status;
- is visiting the study-selected health facility during the enrollment period
- is willing to be followed up three months post-enrollment.

Intervention: The intervention will be presented at the end of Phase 3 of the study. It will be a male-tailored HEP aimed at improving the uptake of HIVST among men. This will be delivered through face to face personal communication with the use of information education communication materials. We anticipate the HEP to address general information on HIV, transmission, prevention, HIVST, anti-retroviral therapy, and care in a manner that is tailored to men.

Randomization procedure: Simple randomization using a randomization table created by a computer software program will be employed.

Blinding: For the purposes of this study, there will be no masking. 

Data Source: We will employ an interviewer-administered questionnaire to collect data.

Analysis: Proportions will be calculated for the uptake of HIVST for both control and intervention groups. KAP scores will be calculated by determining the average score for each individual for each category of KAPs that will be assessed. Tables and graphs will be used where appropriate to present results. Quantitative variables will be summarized by means and standard deviation (SD). Qualitative variables will be summarized using frequency distribution. The student t-test or analysis of variance (ANOVA) will be used to compare if there are differences in KAP scores between different categories of the factor variables. Linear regression will be utilized to determine the association between KAP scores and quantitative factor variables. Multiple logistic regression will be used to determine the association between the uptake of HIVST and the use of HEP whilst adjusting for other study variables. All statistical tests will be concluded at a 5% level of significance. All statistical analysis will be carried in the Stata version 16 statistical package.

Primary outcome(s): The feasibility of a larger RCT trial will be assessed by recruitment and the uptake of HIVST Uptake of HIVST will be measured as self-reported use of a HIVST kit at follow-up interview. 

Secondary outcome(s): Secondary outcome variables will include HIV diagnosis among participants, linkage to care for men who tested positive for HIV, a repeat test from men who tested negative for HIV, and HIV status disclosure to sexual partner(s) post-HIVST.

Table 3 summarizes the outcomes, exploratory factors, and the methods of analysis for each study objective.

## 3. Data Security

The study will be conducted in accordance with the Helsinki Declaration [48]. Ethical approval to conduct this study was obtained from four relevant ethical committees. Study participants will be required to sign written informed consent prior to enrollment after the aim and objectives of the study have been explained and accepted by the participant, and participants will be provided with a copy of the consent form for future reference. Tape-recorded information and written data collected from participants on questionnaires will be stored appropriately in a lockable cabinet with restricted entry. Data obtained will also be captured in statistical software on a password-protected laptop. The transfer of data across the research team will occur via an encrypted flash drive or a secure file transfer protocol. Information provided by participants will be kept anonymous. Data will be kept anonymous during the study and will be kept strictly confidential in storage for two years after the completion of the study within the University of KwaZulu Natal. During the dissemination of the findings, no identifying data will be made public. Therefore, the researcher will take responsibility to ensure that, even after all information has been collected, sources will never be identified.

## 4. Discussion

The aim of this study is to adapt an HEP for improving the uptake of HIVST by men in Rwanda. To the best of our knowledge, this study is the first of its kind in Rwanda. Given the reported low uptake of HTSs among men in Rwanda, and in Sub-Saharan Africa in general, implementing an effective HEP to improve the uptake of HIVST, a new intervention with reported acceptability among men, may help bridge this gap. The pragmatic approach is designed to provide results that are directly applicable to normal practice.

Several studies have called for the need for the implementation of policies that advocate for men’s health [49,50,51]. Tailored HEPs to improve men’s uptake of HTSs have been recommended [52]. As HIVST is relatively new in Rwanda [11], there is need for the implementation of guidelines and policies that advocate for men’s engagement in this new intervention and for the scaling up thereof [53]. The knowledge obtained by the present study may thus guide HIVST implementation in Rwanda with the potential of being further incorporated into routine practice.

Consistent with the nature of pragmatic trials, we aim to influence policy by providing stakeholders with results that are applicable to routine practice. We intend to disseminate findings of the study through reports, publications in peer-reviewed journals, research meetings, and conference presentations.

Due to constraints of time and resources, as this study is for a doctoral degree with limited findings, a pilot RCT will be used to assess the preliminary effects of the HEP. A pilot study is not a hypothesis testing study. However, a pilot study is a requisite initial step, and results can inform feasibility and identify modifications needed in the design of a larger ensuing hypothesis-testing study. In this study, the feasibility of recruitment, intervention implementation, and retention will be examined. Secondly, due to the nature of the HIVST being conducted privately, study outcomes will be based on self-reported data. Finally, the HIVST uptake is only as important as the number of individuals actively linked to care and treatment services. The proposed study will not actively follow up on participants for linkage to care and treatment services. However, sufficient information on linkage to care post-HIVST will be provided on both enrollment and follow-up visits.

## Figures and Tables

**Table 1:** Consolidated framework for implementation research.

Intervention Characteristics
(a) Intervention source: Perception of key stakeholders about whether HIVST externally or internally developed.
(b) Evidence Strength and Quality: Stakeholders’ perceptions of the quality and validity of evidence supporting the belief that HIVST will have desired outcomes.
(c) Relative advantage: Stakeholders’ perception of the advantage of implementing HIVST versus an alternative solution
(d) Adaptability: The degree to which HIVST can be adapted, tailored, refined, or reinvented to meet local needs
(e) Trialability: The ability to test HIVST on a small scale in the organization, and to be able to reverse course (undo implementation) if warranted.
(f) Complexity Perceived difficulty of implementation, reflected by duration, scope, radicalness, disruptiveness, centrality, and intricacy and number of steps required to implement
(g) Design Quality and Packaging Perceived excellence in how HIVST bundled, presented, and assembled
(h) Cost: Costs of HIVST and costs associated with implementing HIVST including investment, supply, and opportunity costs.

**Table 2:** Calculation of sample size and determination of sampling strategy per study site.

Study Site	Number of Monthly Male Clinic Attendees Per Month	Calculated Sample Size for Study Site (Probability Proportionate to Size)	Systematic Random Sampling Strategy
1	400	(400/900 × 384) 170	(900/400) every 2nd person
2	300	(300/900 × 384) 128	(900/300) every 3rd person
3	200	(200/900 × 384) 86	(900/200) every 5th person
Totals	900	384	

**Table 3:** Outcomes, exploratory factors, and the methods of analysis for each study objective.

Outcome Measures Variable	Criteria for Success/Hypothesis	Exploratory Factors	Methods of Analysis
**Phase 1: Objective 1 To conduct a scoping review on health education programs (HEPs) for men in low- and middle-income countries (LMICs)**			
Improved availability, acceptability, and uptake of health education programs (HEPs) by men in low- and middle-income countries (LMICs).	N/A	Availability, acceptability, and uptake of HEPs.	Thematic content analysis Number studies of reporting outcomes of interest.
**Phase 2: Objective 2 To determine key stakeholder and health care providers’ perceptions on implementation of HIVST in Rwanda**			
Perception	N/A	Key stakeholders and health care provider’s perceptions on the implementation of HIV-self testing	Qualitative Thematic content analysis
**Phase 3: Objective 3 To assess HIVST awareness among men in Rwanda**			
Awareness	N/A	Demographic characteristics, knowledge, attitudes, HIV risk perception, and health seeking behavior characteristics	Descriptive statistics or estimates based on 95% confidence intervals (CI) Logistic regression
Acceptability			
**Objective 4 To develop and optimize an HEP for improving uptake of HIVST among men in Rwanda**			
Adapted HEP	N/A	N/A	Nominal group technique
**Phase 4: Objective 5 To determine preliminary impact of the HEP on uptake of HIVST among men in Rwanda**			
- Recruitment - Uptake (self-reported use of the HIVST kit)	- 60 men followed after 3 months - At least 80% will take up HIVST	- Percentage response rate - Proportion of men who will take up HIVST	Descriptive statistics or estimates based on 95% confidence intervals (CI)
% of men who get HIV diagnosis	Higher proportion of HIV diagnosis, linkage to care, and repeat testing for negative participants in the intervention group (HEP), compared with the non-intervention group.	Age, education level, religious belief, sexual behavior, sexual preference, marital status; age of first sexual encounter; the number of sexual partners, the history of STI, circumcision, condom use, alcohol and drug use, perceived risk of contracting HIV, level of income, distance to the point of sale of HIVST	Logistic regression
% of men who tested HIV positive and linked to care			
% of repeat test from men who tested HIV negative			

## Data Availability

Data sharing is not applicable to this article, as it is a study protocol. No datasets were generated or analyzed during the current study.

## Supplementary Materials

The following are available online at https://www.mdpi.com/1010-660X/56/4/149/s1.

## Abbreviations

CFIR	The Consolidated Framework for Implementation Research
HEP	health education program
HIVST	HIV self-testing
HTS	HIV testing service
LMICs	low- and middle-income countries
