# Supplementary material for: Adaptation of a Health Education Program for Improving the Uptake of HIV Self-Testing by Men in Rwanda: A Study Protocol
Source: Medicina (Kaunas). 2020 Mar 26;56(4):149. doi: 10.3390/medicina56040149 (PMC7230617; doi:10.3390/medicina56040149)
Supplement: Supplementary file 1 [file medicina-56-00149-s001.pdf]

Study Title: Adaptation of health education program for improving the uptake of HIV self-testing by men in Rwanda

Supplementary File 1: Interview Guide

Supplementary File 1: Table 1- Ministry of Health/ Rwanda Biomedical Center and NGO stakeholders' in-depth interview

Question	Remarks
Introduction: "Hello, my name is XXX. I will conduct the interview, record and take notes. I invited you to discuss HIV self-testing and your perception on implementation of HIV self- testing in Rwanda. I will ask you several open questions. Your personal opinions and views are very important for us. There are no right or wrong answers. Please feel welcome to express yourself freely during the interview.<br>This conversation will be recorded on tape. This is only for purpose of the research, only the lead researcher indicated on the consent form (and I) will listen to the tape. No names or personal information will be used in the report.<br><br>The interview will last for about forty minutes. Is everything clear about the course of the interview?	Before starting the interview all participants will be informed about the purpose of the discussion, confidentiality and practical issues.
1. Please tell me what you know about HIV self-testing in Rwanda	For acquaintance with the participants and gradually focusing the discussion on the study topic
2. Are you involved in HIV self-testing implementation in Rwanda?<br><br>*If YES what is your current role? If NO what will be your role.*	To probe information on participant's role in implementation of HIVST in Rwanda.
3. What is your perception toward current HIV testing services in Rwanda? What has worked? What still needs to be strengthened?<br><br>*Current HIV testing services structure in Rwanda at different levels*<br>*Perceived challenges with current HIV testing services in Rwanda*<br>*Perceptions on system readiness for HIVST implementation in Rwanda*<br>*Perception on how HIVST will complement current HIV testing services*	Introduction question aims to explore participant perspective on current HIV testing services.<br>The sub-questions to the introduction question will be aimed to assess perceptions toward current organizational characteristics, culture, implementation climate and readiness

4. Given your experience with HIV testing serves in Rwanda, what do you think will need to be set up before HIV self-testing can be rolled up at large scale successfully?<br><br>*Adaptability of HIVST in intervention in Rwanda*<br>*Complexity of HIVST implementation in Rwanda*<br>*Resources currently available for HIVST implementation in Rwanda and perceived shortfalls*<br>*Perceptions on costs associated with HIVST implementation in Rwanda for government and for the users*	Introduction question aims to explore participant perspective on adaptability of HIVST in Rwanda.<br>The sub-questions to the introduction question will be aimed to assess perceptions toward complexity, resources and cost of HIVST implementation.
5. What are your perceptions on the existing HIV testing policies and what regulatory challenges do you perceive for HIVST policy?	To assess participant perception on the existing HIV testing policies and perceived regulatory challenges with HIVST policy.<br>The sub-questions to the introduction question will aim to assess more information on stated potential challenges.
6. What are your general impressions on HIV self-testing?	To explore positive and negative perceptions toward HIV self-testing.<br><br>Sub- questions will be used to explore the positive and negative perceptions toward HIV self-testing based on the participant response.
7. In conclusion, what is your recommendation on how Ministry of Health should implement HIV self-testing in Rwanda to ensure uptake and increase use of HIV testing services?	This question will be used to further explore any additional perceptions on implementation. Recommendations will be explored from the participants on what they feel would make HIVST implantation a success in Rwanda.<br>At the end of the interview, the participants will be given the

	opportunity to add remarks or suggestions related to the subject topic.

Supplementary File 1: Table 2 - Health care provider (HIV Clinic nurse/ counsellor) in-depth interview

Question	Remarks
Introduction: "Hello, my name is XXX. I will conduct the interview, record and take notes. I invited you to discuss HIV self-testing and your perception on implementation of HIV self- testing in Rwanda. I will ask you several open questions. Your personal opinions and views are very important for us. There are no right or wrong answers. Please feel welcome to express yourself freely during the interview.<br>This conversation will be recorded on tape. This is only for purpose of the research, only the lead researcher indicated on the consent form (and I) will listen to the tape. No names or personal information will be used in the report.<br><br>The interview will last for about forty minutes. Is everything clear about the course of the interview?	Before starting the interview all participants will be informed about the purpose of the discussion, confidentiality and practical issues.
1. Please tell me what you know about HIV self-testing in Rwanda	For acquaintance with the participants and gradually focusing the discussion on the study topic
2. Are you involved in HIV self-testing implementation in Rwanda?<br><br>*If YES what is your current role? If NO what will be your role.*	To probe information on participant's role in implementation of HIVST in Rwanda.
3. What is your perception toward current HIV testing services in Rwanda? What has worked? What still needs to be strengthened?<br><br>*Current HIV testing services structure in Rwanda at different levels*<br>*Perceived challenges with current HIV testing services in Rwanda*<br>*Perceptions on system readiness for HIVST implementation in Rwanda*<br>*Perception on how HIVST will complement current HIV testing services*	Introduction question aims to explore participant perspective on current HIV testing services.<br>The sub-questions to the introduction question will be aimed to assess perceptions toward current organizational characteristics, culture,

	implementation climate and readiness
4. Given your experience with HIV testing serves in Rwanda, what do you think will need to be set up before HIV self-testing can be rolled up at large scale successfully?<br><br>*Adaptability of HIVST in intervention in Rwanda*<br>*Complexity of HIVST implementation in Rwanda*<br>*Resources currently available for HIVST implementation in Rwanda and perceived shortfalls*<br>*Perceptions on costs associated with HIVST implementation in Rwanda for government and for the users*	Introduction question aims to explore participant perspective on adaptability of HIVST in Rwanda.<br>The sub-questions to the introduction question will be aimed to assess perceptions toward complexity, resources and cost of HIVST implementation.
5. What are your perceptions on the existing HIV testing policies and what regulatory challenges do you perceive for HIVST policy?	To assess participant perception on the existing HIV testing policies and perceived regulatory challenges with HIVST policy.<br>The sub-questions to the introduction question will aim to assess more information on stated potential challenges.
6. What are your general impressions on HIV self-testing?	To explore positive and negative perceptions toward HIV self-testing.<br>Sub- questions will be used to explore the positive and negative perceptions toward HIV self-testing based on the participant response.
7. What are the anticipated challenges with HIV self-testing uptake among men in your district?	To assess health care provider perceived barriers to uptake of HIVST by men
8. In conclusion, what is your recommendation on how Ministry of Health should implement HIV self-testing in Rwanda to ensure uptake and increase use of HIV testing services?	This question will be used to further explore any additional perceptions on implementation. Recommendations will be explored from the participants on what they feel would make HIVST implantation a success in Rwanda.

	At the end of the interview, the participants will be given the opportunity to add remarks or suggestions related to the subject topic.

Supplementary File 1: Table 3 - National Reference Laboratory staff in-depth interview

Question	Remarks
Introduction: "Hello, my name is XXX. I will conduct the interview, record and take notes. I invited you to discuss HIV self-testing and your perception on implementation of HIV self- testing in Rwanda. I will ask you several open questions. Your personal opinions and views are very important for us. There are no right or wrong answers. Please feel welcome to express yourself freely during the interview.<br>This conversation will be recorded on tape. This is only for purpose of the research, only the lead researcher indicated on the consent form (and I) will listen to the tape. No names or personal information will be used in the report.<br>The interview will last for about forty minutes. Is everything clear about the course of the interview?	Before starting the interview all participants will be informed about the purpose of the discussion, confidentiality and practical issues.
1. Please tell me what you know about HIV self-testing in Rwanda	For acquaintance with the participants and gradually focusing the discussion on the study topic
2. Are you involved in HIV self-testing implementation in Rwanda?<br>*If YES what is your current role? If NO what will be your role*	To probe information on participant's role in implementation of HIVST in Rwanda.
3. What is your perception toward current HIV testing services in Rwanda? What has worked? What still needs to be strengthened?<br><br>*Current HIV testing services structure in Rwanda at different levels*<br>*Perceived challenges with current HIV testing services in Rwanda*<br>*Perceptions on system readiness for HIVST implementation in Rwanda*<br>*Perception on how HIVST will complement current HIV testing services*	Introduction question aims to explore participant perspective on current HIV testing services.<br>The sub-questions to the introduction question will be aimed to assess perceptions toward current organizational characteristics, culture,

	implementation climate and readiness
4. Given your experience with HIV testing serves in Rwanda, what do you think will need to be set up before HIV self-testing can be rolled up at large scale successfully?<br><br>*Adaptability of HIVST in intervention in Rwanda*<br>*Complexity of HIVST implementation in Rwanda*<br>*Resources currently available for HIVST implementation in Rwanda and perceived shortfalls*<br>*Perceptions on costs associated with HIVST implementation in Rwanda for government and for the users*	Introduction question aims to explore participant perspective on adaptability of HIVST in Rwanda.<br>The sub-questions to the introduction question will be aimed to assess perceptions toward complexity, resources and cost of HIVST implementation.
5. What are your perceptions on the existing HIV testing policies and what regulatory challenges do you perceive for HIVST policy?	To assess participant perception on the existing HIV testing policies and perceived regulatory challenges with HIVST policy.<br>The sub-questions to the introduction question will aim to assess more information on stated potential challenges.
6. What are your general impressions on HIV self-testing?	To explore positive and negative perceptions toward HIV self-testing.<br><br>Sub- questions will be used to explore the positive and negative perceptions toward HIV self-testing based on the participant response.
7. What are the anticipated challenges with HIV self-testing from the laboratory perspective?	To assess laboratory perceived challenges to implementation of HIVST
8. In conclusion, what is your recommendation on how Ministry of Health should implement HIV self-testing in Rwanda to ensure uptake and increase use of HIV testing services?	This question will be used to further explore any additional perceptions on implementation. Recommendations will be explored from the participants on what they feel would make HIVST implantation a success in Rwanda.

	At the end of the interview, the participants will be given the opportunity to add remarks or suggestions related to the subject topic.

Supplementary File 1: Table 4 - RBC/Medical and Pharmaceutical Products Department (supply chain) staff in-depth interview

Question	Remarks
Introduction: "Hello, my name is XXX. I will conduct the interview, record and take notes. I invited you to discuss HIV self-testing and your perception on implementation of HIV self- testing in Rwanda. I will ask you several open questions. Your personal opinions and views are very important for us. There are no right or wrong answers. Please feel welcome to express yourself freely during the interview.<br>This conversation will be recorded on tape. This is only for purpose of the research, only the lead researcher indicated on the consent form (and I) will listen to the tape. No names or personal information will be used in the report.<br><br>The interview will last for about forty minutes. Is everything clear about the course of the interview?	Before starting the interview all participants will be informed about the purpose of the discussion, confidentiality and practical issues.
1. Please tell me what you know about HIV self-testing in Rwanda	For acquaintance with the participants and gradually focusing the discussion on the study topic
2. Are you involved in HIV self-testing implementation in Rwanda?<br><br>*If YES what is your current role? If NO what will be your role.*	To probe information on participant's role in implementation of HIVST in Rwanda.
3. What is your perception toward current HIV testing services in Rwanda? What has worked? What still needs to be strengthened?<br><br>*Current HIV testing services structure in Rwanda at different levels*<br>*Perceived challenges with current HIV testing services in Rwanda*	Introduction question aims to explore participant perspective on current HIV testing services.<br>The sub-questions to the introduction question will be aimed to assess

Perceptions on system readiness for HIVST implementation in Rwanda<br>*Perception on how HIVST will complement current HIV testing services*	perceptions toward current organizational characteristics, culture, implementation climate and readiness
4. Given your experience with HIV testing serves in Rwanda, what do you think will need to be set up before HIV self-testing can be rolled up at large scale successfully?<br><br>*Adaptability of HIVST in intervention in Rwanda*<br>*Complexity of HIVST implementation in Rwanda*<br>*Resources currently available for HIVST implementation in Rwanda and perceived shortfalls*<br>*Perceptions on costs associated with HIVST implementation in Rwanda for government and for the users*	Introduction question aims to explore participant perspective on adaptability of HIVST in Rwanda.<br>The sub-questions to the introduction question will be aimed to assess perceptions toward complexity, resources and cost of HIVST implementation.
5. What are your perceptions on the existing HIV testing policies and what regulatory challenges do you perceive for HIVST policy?	To assess participant perception on the existing HIV testing policies and perceived regulatory challenges with HIVST policy.<br>The sub-questions to the introduction question will aim to assess more information on stated potential challenges.
6. What are your general impressions on HIV self-testing?	To explore positive and negative perceptions toward HIV self-testing.<br>Sub- questions will be used to explore the positive and negative perceptions toward HIV self-testing based on the participant response.
7. What measure need to be put in place prior to implementation of HIV self-testing from the supply chain perspective?	To assess supply chain manager perception on measures to be put in place prior to implementation of HIVST.
8. What are the anticipated challenges with implementation of HIV self-testing from the supply chain perspective?	To assess supply chain manager perceived challenges to implementation of HIVST

9. In conclusion, what is your recommendation on how Ministry of Health should implement HIV self-testing in Rwanda to ensure uptake and increase use of HIV testing services?	This question will be used to further explore any additional perceptions on implementation. Recommendations will be explored from the participants on what they feel would make HIVST implantation a success in Rwanda.<br>At the end of the interview, the participants will be given the opportunity to add remarks or suggestions related to the subject topic.

Supplementary File 2: QUESTIONNAIRE

Respondent study ID: ____________________

Thank you for agreeing to participate in this study. The first sets of questions are about your lifestyle, followed by questions related to HIV- self testing knowledge and perception. Mark with an X in the appropriate box. N/A (not applicable)

PART I: DEMOGRAPHIC INFORMATION

1. Gender:

Male
☐

2. Age: ___
3. What is your level of education? (Tick one)

Primary	Secondary	University	Don't know
☐	☐	☐	☐

4. Source of financial income:

Unemployed	Professional	Self-employed
☐	☐	☐

5. Location in which you reside:

Gasabo	Nyarugenge	Kicukiro
☐	☐	☐

6. Marital status:

Single	Married	Cohabitation	Widowed	Separated
☐	☐	☐	☐	☐

7. Age at marriage/cohabitating with your spouse or partner: ____

8. What is your sexual preference? (Tick one):

Heterosexual	Homosexual	Bisexual
☐	☐	☐

PART II: HEALTH STATUS AND SEXUAL BEHAVIOUR

1. Have you ever had sexual intercourse? (Tick one)

Yes	No
☐	☐

2. If you answered Yes to question 1 above, how old were you then? ____

3. How many sexual partners have you had in your life? ______

4. In the last 12 months, with how many different sexual partners did you have? ____

5. If married/cohabitating, how many different sexual partners ever had? ___

6. Have you ever paid or received payment for sex (payment includes monetary or non-monetary gifts)?

Yes	No
☐	☐

7. Have you ever paid or received payment for sex (payment includes monetary or non-monetary gifts) in the last one month? (Tick one)

Yes	No
☐	☐

8. Have you ever had a sexually transmitted infection? (Tick one)

Yes	No
☐	☐

9. If Yes to 8 above, what was the STI(s)? ______________________

10. Are you circumcised? (Tick one)

Yes	No
☐	☐

11. How often did your sexual partners ever shared their HIV status with you?

In the last ten sexual encounters; 4 scale response for number of times your sexual partners have shared their HIV status with you. Never being zero and always being each time. Rarely – 1-5 last times; Sometimes 6-9 times

Never	Rarely	Sometimes	Always
☐	☐	☐	☐

12. You know how to use a condom (Tick one)

Strongly Disagree	Disagree	Neutral	Agree	Strongly Agree
☐	☐	☐	☐	☐

13. The first time you had sex, did you use a condom? (Tick one)

Yes	No
☐	☐

14. How often did you or any of your partner ever used a condom in any of your sexual intercourse?
In the last ten sexual encounters; 4 scale response for number of times you used a condom. Never being zero and always being each time. Rarely – 1-5 last times; Sometimes 6-9 times

Never	Rarely	Sometimes	Always
☐	☐	☐	☐

15. How often did you receive any condoms in the last twelve months?

In the last ten sexual encounters; 4 scale response for number of times you received or bought a condom. Never being zero and always being each time. Rarely – 1-5 last times; Sometimes 6-9 times

Never	Rarely	Sometimes	Always
☐	☐	☐	☐

16. How often did you use a condom in the last twelve months?
    In the last twelve months (1 year). On average, in your sexual encounters; 1 scale response for number of times you received or bought a condom. Never being zero and always being each time. Rarely – 1-5 (less than half the times); Sometimes 6-9 (more than half the times)

Never	Rarely	Sometimes	Always
☐	☐	☐	☐

17. How often do you take any alcohol or use any drugs?
    In the last one month; 1 scale response for number of times you consumed alcohol or used drugs. Never being zero and always being every day. Rarely – 1-5 times; Sometimes 6-9 times

Never	Rarely	Sometimes	Always
☐	☐	☐	☐

18. How often do you indulge in any sexual activities after taking alcohol or drugs?

    In the last ten times you were drunk; 4 scale response for number of times you engaged in sex. Never being zero and always being every day. Rarely – 1-5 times; Sometimes 6-9 times

Never	Rarely	Sometimes	Always
☐	☐	☐	☐

19. When you have sex after drinking and/or take drugs, how often do you use a condom?

In the last ten times you had sex when drunk or after drugs; 4 scale response for number of times you engaged in sex. Never being zero and always being every day. Rarely – 1-5 times; Sometimes 6-9 times

Never	Rarely	Sometimes	Always
☐	☐	☐	☐

20. How often have you had sex after alcohol or drugs?

In the last ten times you were drunk; 4 scale response for number of times you engaged in sex after alcohol or drugs. Never being zero and always being every day. Rarely – 1-5 times; Sometimes 6-9 times

Never	Rarely	Sometimes	Always
☐	☐	☐	☐

21. How often have you ever paid for sex while under the influence of drugs or alcohol?

In the last ten times you were drunk; 4 scale response for number of times you paid for sex. Never being zero and always being every day. Rarely – 1-5 times; Sometimes 6-9 times

Never	Rarely	Sometimes	Always
☐	☐	☐	☐

22. To what extent do you think you are at risk of acquiring HIV?

Very High	High	Fairly High	Low	Very Low
☐	☐	☐	☐	☐

PART III: HEALTH SEEKING BEHAVIOUR

Mark with an X in the appropriate box.

23. What is your reason for visiting the health facility today?

Accompany a relative	Accompany my spouse	Visiting a relative who is admitted	To seek health services

☐	☐	☐	☐

24. On average, how often have you been sick in the last three months?

Never	1 -3 times	4 - 5 times	More than 5 times
☐	☐	☐	☐

25. The last three times you were sick, which of the following did you do first?

1. Consultation with qualified medical practitioners	☐
2. Consultation with community health worker	☐
3. Consultation with traditional health care practitioners	☐
4. Consultation with over the counter drug sellers	☐
5. Consultation with self and other family members	☐
6. Nothing	☐

26. When was the last time you visited a health facility or professional medical practitioner for an ordinary check-up?

Never	1–3 months ago	4–6 months ago	6–12 months ago	More than a year ago
☐	☐	☐	☐	☐

PART IV: KNOWLEDGE OF HIV SELF TESTING

Mark with an X in the appropriate box. N/A (not applicable)

1. Do you know about HIV self- testing?

Yes	No
☐	☐

2. If you answered Yes to question 1 above, where did you learn about it? ______

3. HIV self-testing kits are available in Rwanda

Strongly Disagree	Disagree	Neutral	Agree	Strongly Agree
☐	☐	☐	☐	☐

4. HIV self-testing may use saliva to test for HIV

Strongly Disagree	Disagree	Neutral	Agree	Strongly Agree
☐	☐	☐	☐	☐

5. One testing device can be re-used

Strongly Disagree	Disagree	Neutral	Agree	Strongly Agree
☐	☐	☐	☐	☐

6. HIV self-testing cannot be performed using blood

Strongly Disagree	Disagree	Neutral	Agree	Strongly Agree
☐	☐	☐	☐	☐

7. HIV self-testing may use urine to test for HIV

Strongly Disagree	Disagree	Neutral	Agree	Strongly Agree
☐	☐	☐	☐	☐

8. HIV self-testing kits do not expire

Strongly Disagree	Disagree	Neutral	Agree	Strongly Agree
☐	☐	☐	☐	☐

9. HIV self-testing results may be ready in one minute for some tests

Strongly Disagree	Disagree	Neutral	Agree	Strongly Agree

☐	☐	☐	☐	☐

10. Some HIV self-testing kits are over 99% accurate

Strongly Disagree	Disagree	Neutral	Agree	Strongly Agree
☐	☐	☐	☐	☐

PART V: ATTITUDE AND PERCEPTION TOWARD HIV SELF TESTING

1. As of 20 February 2017, it is recommended by Ministry of Health to use HIV self-testing kits as an additional strategy to reach out to people who are not yet tested and there are plans for a national roll out in 2019. Do you agree with this recommendation?

Strongly Disagree	Disagree	Neutral	Agree	Strongly Agree
☐	☐	☐	☐	☐

2. It is good to recommend HIV self-testing to a friend.

Strongly Disagree	Disagree	Neutral	Agree	Strongly Agree
☐	☐	☐	☐	☐

3. Would you recommend HIV self-testing to your sexual partner

Strongly Disagree	Disagree	Neutral	Agree	Strongly Agree
☐	☐	☐	☐	☐

4. Good to disclose your HIV self-testing results to your sexual partner

Strongly Disagree	Disagree	Neutral	Agree	Strongly Agree
☐	☐	☐	☐	☐

5. Do you agree with HIV testing without pre- or immediate post-test counselling?

Strongly Disagree	Disagree	Neutral	Agree	Strongly Agree
☐	☐	☐	☐	☐

6. How do you agree HIV results from saliva?

Strongly Disagree	Disagree	Neutral	Agree	Strongly Agree
☐	☐	☐	☐	☐

7. Will you go seeking treatment and care if you test HIV positive using HIV self - testing?

Strongly Disagree	Disagree	Neutral	Agree	Strongly Agree
☐	☐	☐	☐	☐

8. How soon will you seek treatment and care at a health facility after testing positive with HIV self- testing?

Never	Less than a year	Less than a month	Less than a week	Same day
☐	☐	☐	☐	☐

9. How frequent will you perform HIV self-testing if you test negative the first time?

Never	Rarely	Sometimes	Always
☐	☐	☐	☐

10. What factor do you think will contribute the most to men not using HIV self-testing? (Tick one)

Lack of knowledge about it	☐
Instructions not clear	☐
No money to buy	☐
No pre- and post- test counselling	☐

Other (Specify)	
